# Time is Brain: The Future for Acute Ischemic Stroke Management is the Utilization of Steerable Microcatheters for Reperfusion

**DOI:** 10.7759/cureus.3842

**Published:** 2019-01-07

**Authors:** Taylor S Harmon, Paul C Hulsberg, Joseph R McFarland, Victoria V Villescas, Jerry Matteo

**Affiliations:** 1 Radiology, University of Texas Medical Branch, Galveston, USA; 2 Interventional Radiology, University of Florida College of Medicine, Jacksonville, USA; 3 Interventional Radiology, The University of Texas Medical Branch, Galveston, USA; 4 Diagnostic Radiology, University of Florida College of Medicine, Jacksonville, USA; 5 Radiology, University of Florida College of Medicine, Jacksonville, USA

**Keywords:** steerable microcatheter, interventional radiology, mechanical thrombectomy, stent retriever, stroke, computed tomography angiography, computed tomography perfusion, penumbra, tissue plasminogen activator, reperfusion catheter

## Abstract

Stroke is the fifth leading cause of death in the United States and is one of the leading causes of patient disability. Treatments for intracranial intravascular damage as a result of stroke have evolved extensively over recent decades, as management has become increasingly innovative. Various prospective studies and years of data have refined the current guidelines for treatment of acute ischemic stroke (AIS) and also reflect on the novel interventions for stroke management. Nonetheless, AIS remains a difficult and multifactorial etiology of disease to treat. As physicians adapt evidence-based knowledge to their interventional management of patients with AIS, the accompanied use of intravascular devices, such as steerable microcatheters, reduces radiation and procedure time. Considering all of the applications for steerable microcatheters, the use of these devices for AIS interventions may be most necessary.

## Introduction

The beginning of mechanical and pharmacological thrombectomy in the treatment of acute ischemic stroke (AIS) originates with the evidence-based use of intravenous recombinant tissue plasminogen activator (rtPA). In 1995, the National Institute of Neurological Disorders and Stroke (NINDS) sponsored two randomized clinical trials that reviewed 624 patients presenting with symptoms of AIS within three hours of onset [1-2]. The retrospective study concluded that there was a 16 percent increase in favorable outcome at three months (modified Rankin scale: 0-1) [2]. Although the study revealed an increase in intracranial hemorrhage, the patient outcomes led the Food and Drug Administration (FDA) to approve intravenous rtPA for treatment of patients with AIS presenting within three hours of onset [1-2].

Following the NINDS-sponsored retrospective study in 1995, the subsequent European Cooperative Acute Stroke Study III (ECASS-3) randomized a clinical trial of 821 patients affected with moderate to severe AIS symptoms, which was conducted to show the benefits of intravenous rtPA administered within three and four and one-half hours [3]. The results of the study showed compelling evidence that patients presenting with AIS treated within four and one-half hours, and more especially within three hours, benefited the most from rtPA therapy [1,3]. Furthermore, since the NINDS and ECASS-3 retrospective trials, other studies with immense power have shown that treatment of AIS with intravenous rtPA beyond four and one-half hours is not beneficial [1,4-6].

Currently, all practicing stroke services will not treat AIS with intravenous rtPA past the allotted four and one-half hours from symptom onset. However, evidence-based literature suggests that patients who receive intravenous rtPA alone only reperfuse 10 to 15 percent of internal carotid artery occlusions and 25 to 50 percent of proximal middle cerebral artery occlusions, resulting in only 35 to 40 percent of positive patient prognosis (such as functional dependence) [1,7-8]. As a result of the large disparity between adequately and inadequately treated AIS patients from rtPA alone, the use of mechanical thrombectomy devices have been developed and are presently used to further manage these patients.

Coil retriever and aspiration devices were first approved by the FDA based on a series of single group studies demonstrating statistically significant evidence for proximal artery reperfusion, when interventional operators employed mechanical thrombectomy [1]. The Penumbra® Pivotal Stroke Trial and Mechanical Thrombectomy for Acute Ischemic Stroke (MERCI) trial were among the first studies to prove that mechanical thrombectomy was superior for certain patients with AIS over intravenous rtPA alone, more particularly in patients with intracranial proximal arterial occlusions [9-11].

Since the Penumbra® and MERCI trials, more evidence-based studies have proven that mechanical and intra-arterial treatments for AIS are superior to intravenous rtPA therapy. Four foundational randomized clinical trials have shown that stent-retriever devices are more beneficial for the treatment of patients with AIS against the standard intravenous rtPA therapy [12-13]. Additionally, the Multicenter Randomized Clinical Trial of Endovascular Treatment for Acute Ischemic Stroke (MR CLEAN) phase-3 provided the first evidence of improved clinical patient prognosis 90 days after AIS symptom onset, when mechanical thrombectomy devices were used within six hours [13]. The Endovascular Treatment for Small Core and Proximal Occlusion Ischemic Stroke trial demonstrated the same results as the MR CLEAN trial but proved that patients with AIS symptoms benefited from mechanical thrombectomy 12 hours after onset [14]. Within the last decade, there has been irrefutable evidence that suggests mechanical thrombectomy is not only superior to systemic intravenous rtPA but that the advancement and development of mechanical devices offer better patient outcomes for those presenting with AIS.

Mechanical thrombectomy devices and reperfusion systems improve patient outcomes and are constantly being redesigned for efficiency. Of the most innovative mechanical devices that are used by neurointerventional operators, the steerable microcatheter might be the most underrated and least discussed. Originally designed for body interventional procedures, the steerable microcatheter offers added maneuverability that a traditional wire and catheter system does not. Small vascular branches that are difficult to access by a wire and catheter model are easily traversed by a steerable microcatheter, which can be directed by the operator from outside the patient. In a setting where the interventional operator highly values time to procedure completion, AIS patients would benefit from a device that significantly decreases time burden. The following case presents a patient with AIS and is quickly intervened upon using traditional neurointerventional devices, in addition to a steerable microcatheter, used to traverse the area of ischemia. The case highlights the efficiency of time to procedure completion and is a testimony to prospectively increasing the functional capabilities of the patient in the post-procedure period.

## Technical report

**Figure 1:**
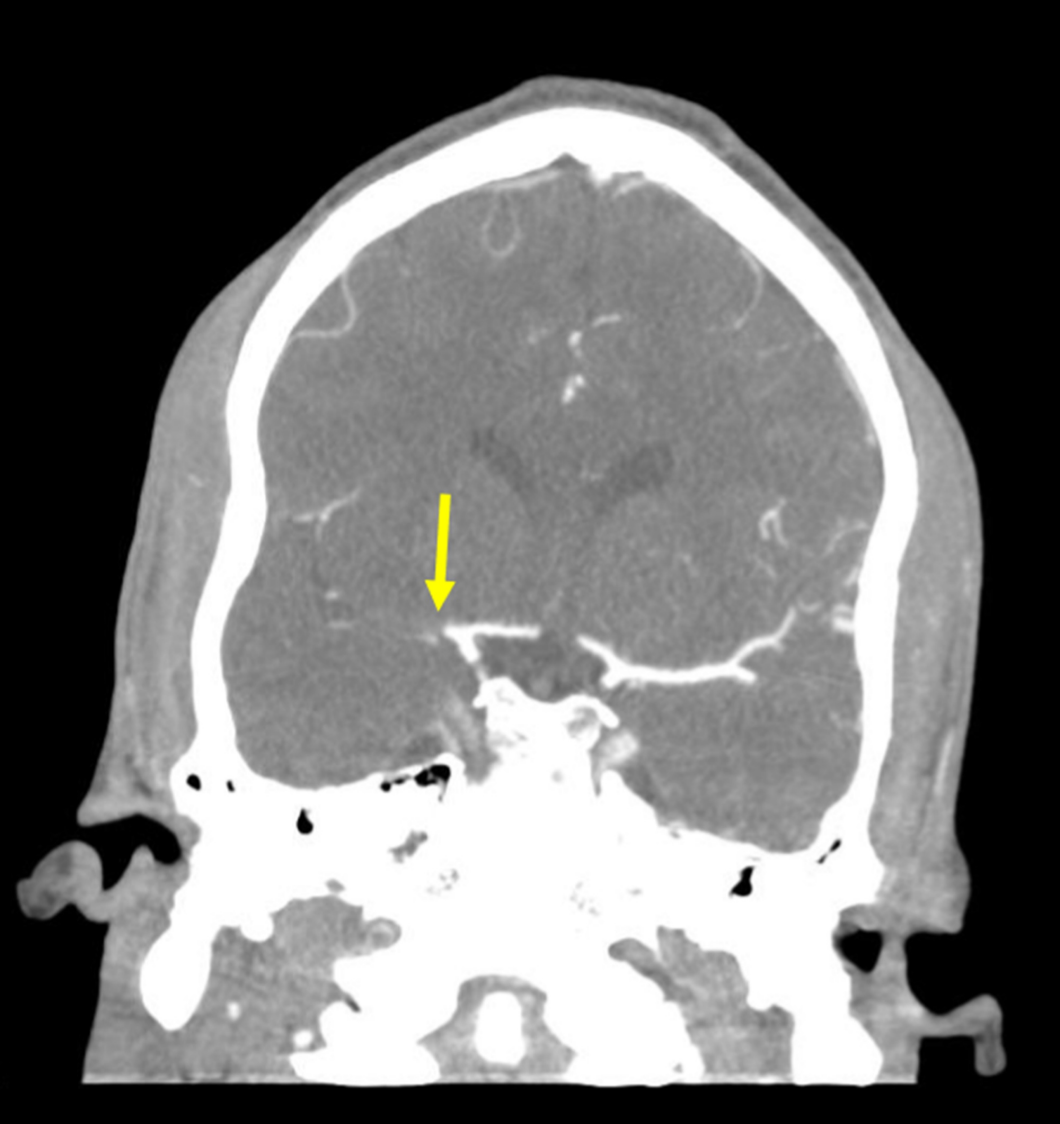
Computed Tomography Angiogram A coronal computed tomography angiogram of the patient's head shows an acute ischemic stroke in the right proximal middle cerebral artery (yellow arrow).

A 29-year-old male presented with signs and symptoms of functional impairment significant of AIS. A non-contrast computed tomography (CT) of the patient’s head was ordered to rule out hemorrhage. A CT angiogram of the patient was then ordered to identify the location of the ischemic event. The CT angiogram revealed AIS within the right proximal M1 territory of the middle cerebral artery (Figure 1).

**Figure 2:**
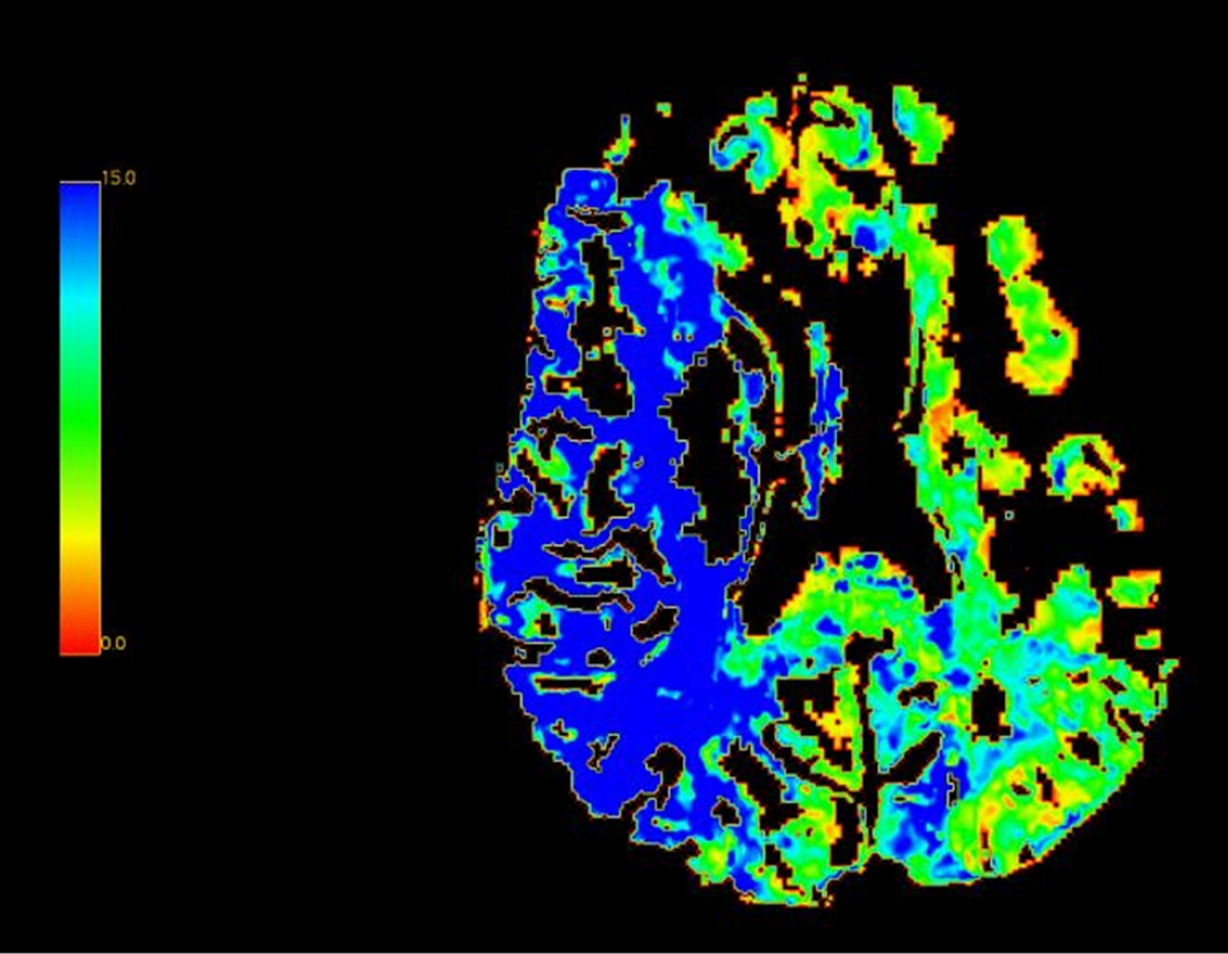
Computed Tomography Perfusion Study A computed tomography perfusion study shows decreased cerebral blood flow within the penumbra territory (right middle cerebral artery), represented by the blue coloration. Otherwise, normal perfusion is indicated by the green coloration.

A CT perfusion scan of the head was then ordered to further identify the core and surrounding penumbra. The CT perfusion scan demonstrated severely diminished cerebral blood volume in the right middle cerebral artery territory, in concordance with the CT angiogram (Figure 2).

**Figure 3:**
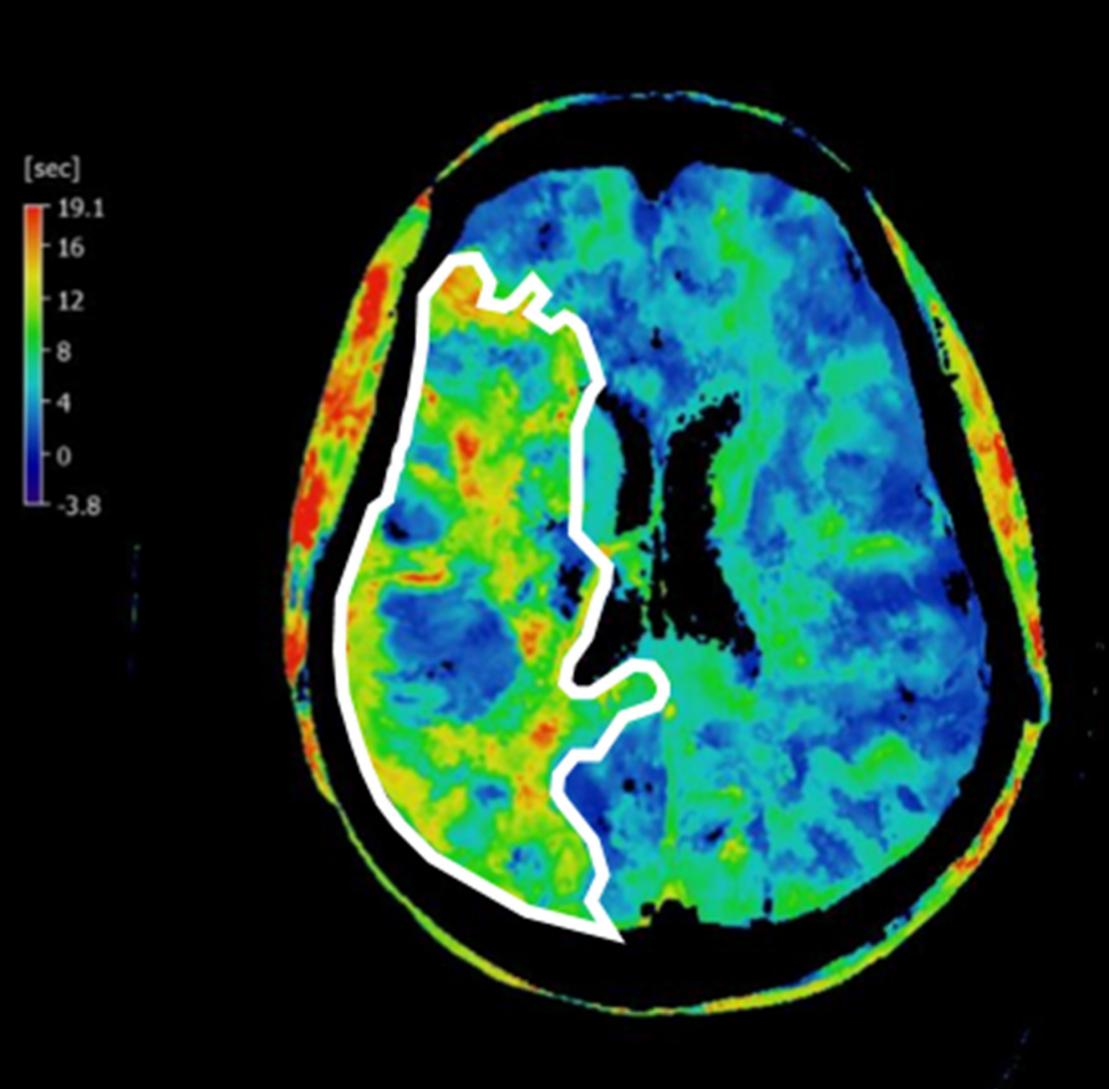
Time to Peak Computed Tomography Perfusion Study A time to peak computed tomography study demonstrates delayed blood flow in the penumbra, within the right middle cerebral artery distribution (white outline).

A corresponding time to peak setting indicates delayed flow within the penumbra, also demonstrated by the CT perfusion scan (Figure 3).

**Figure 4:**
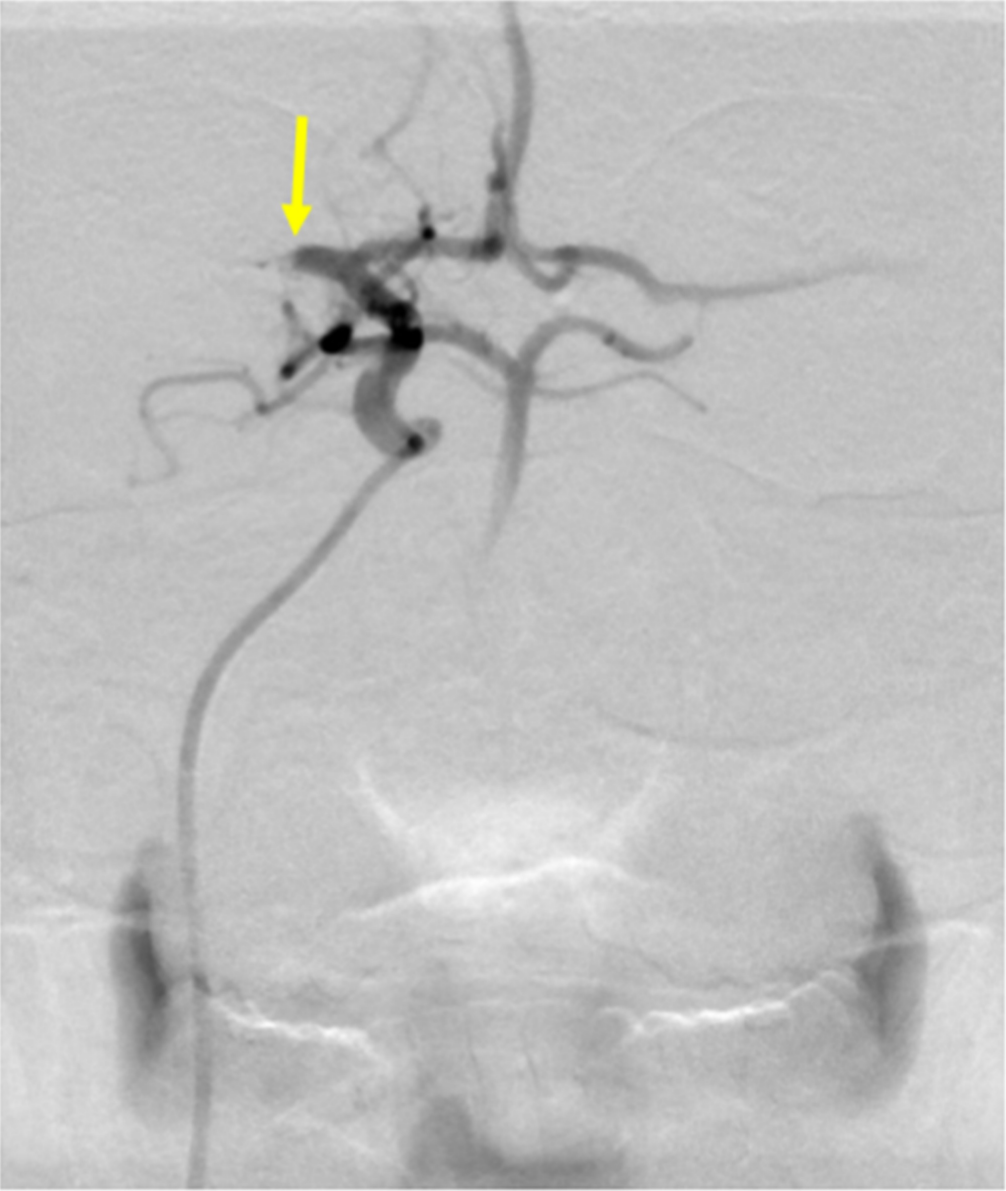
Cerebral Arteriogram of Acute Ischemic Stroke A cerebral arteriogram demonstrates a thrombotic occlusion in the proximal middle cerebral artery (yellow arrow).

The patient was then transported to the biplane neurointerventional suite for further management. The skin over the patient’s right common femoral artery was anesthetized using lidocaine and subsequently accessed using a micropuncture kit. A guidewire was then advanced into the aortic arch. The micropuncture sheath was exchanged for a six French sheath. A cerebral angiogram was performed to visualize the area of occlusion. A filling defect related to thrombus within the right proximal middle cerebral artery was seen (Figure 4).

**Figure 5:**
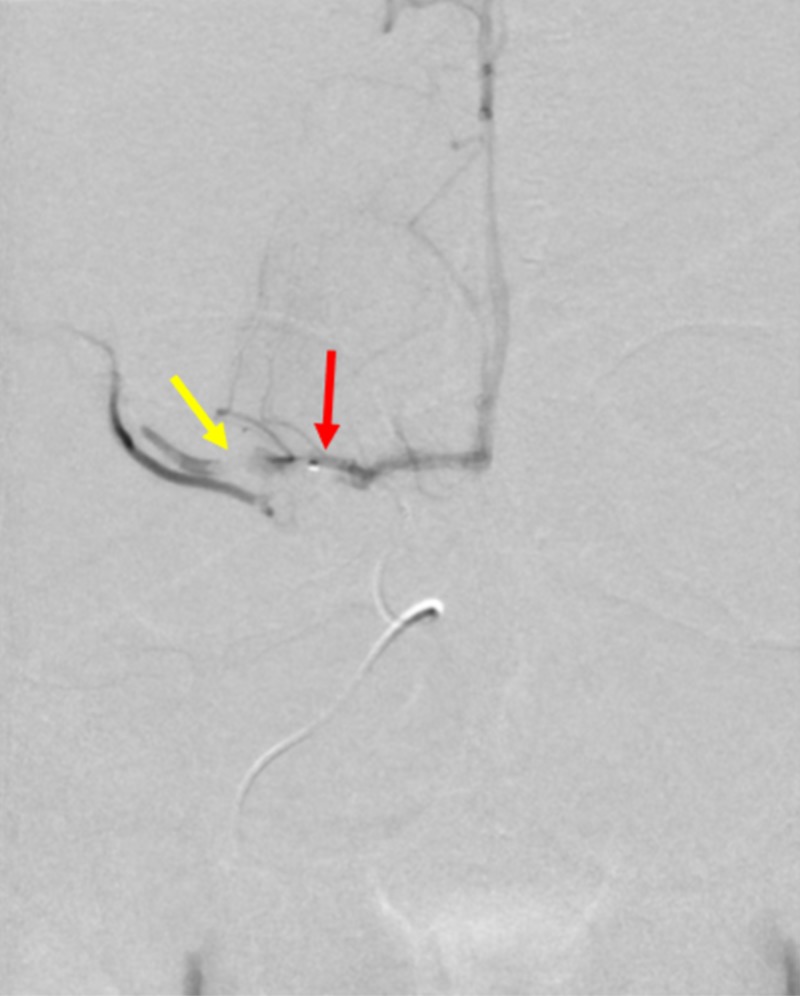
Cerebral Angiogram with a SwiftNINJA® Steerable Microcatheter A cerebral angiogram with a SwiftNINJA® steerable microcatheter (red arrow) demonstrates the area of thrombotic occlusion (yellow arrow) in the right proximal middle cerebral artery.

The right internal carotid artery was selectively catheterized with a JB2 catheter, which was then exchanged for a SwiftNINJA® steerable microcatheter (Merit Medical Systems, South Jordan, Utah; Figure 5).

**Figure 6:**
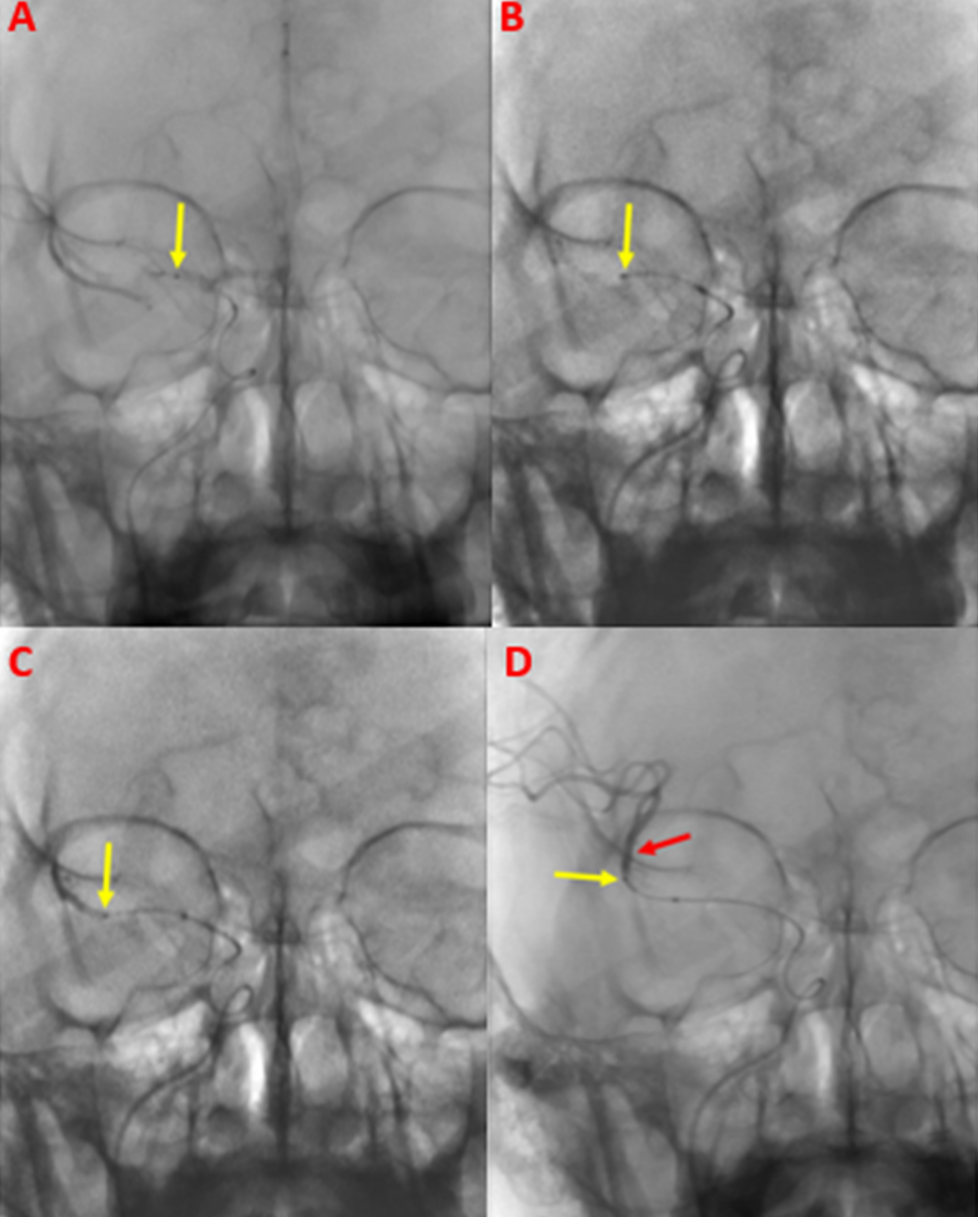
Traversing the Thrombus within the Middle Cerebral Artery Using a SwiftNINJA® Steerable Microcatheter A SwiftNINJA® steerable microcatheter was used to traverse the proximal segment of the patient's right middle cerebral artery. In a stepwise fashion, the SwiftNINJA® steerable microcatheter (yellow arrows) is seen as it is advanced proximally within the middle cerebral artery M1 segment (A), to the midportion (B), and distally (C). The SwiftNINJA® steerable microcatheter was then successfully advanced beyond the thrombus within the right middle cerebral artery, into the M2 segment (D). Once across, the interventional operator administered contrast through the SwiftNINJA® steerable microcatheter (yellow arrow) demonstrating patency of the M2 segment (red arrow).

A SwiftNINJA® steerable microcatheter was used to cross the thrombotic occlusion, without the use of a guidewire. The area of thrombus was quickly crossed with the SwiftNINJA® steerable microcatheter, and the M2 segment of the right middle cerebral artery was catheterized (Figure 6).

**Figure 7:**
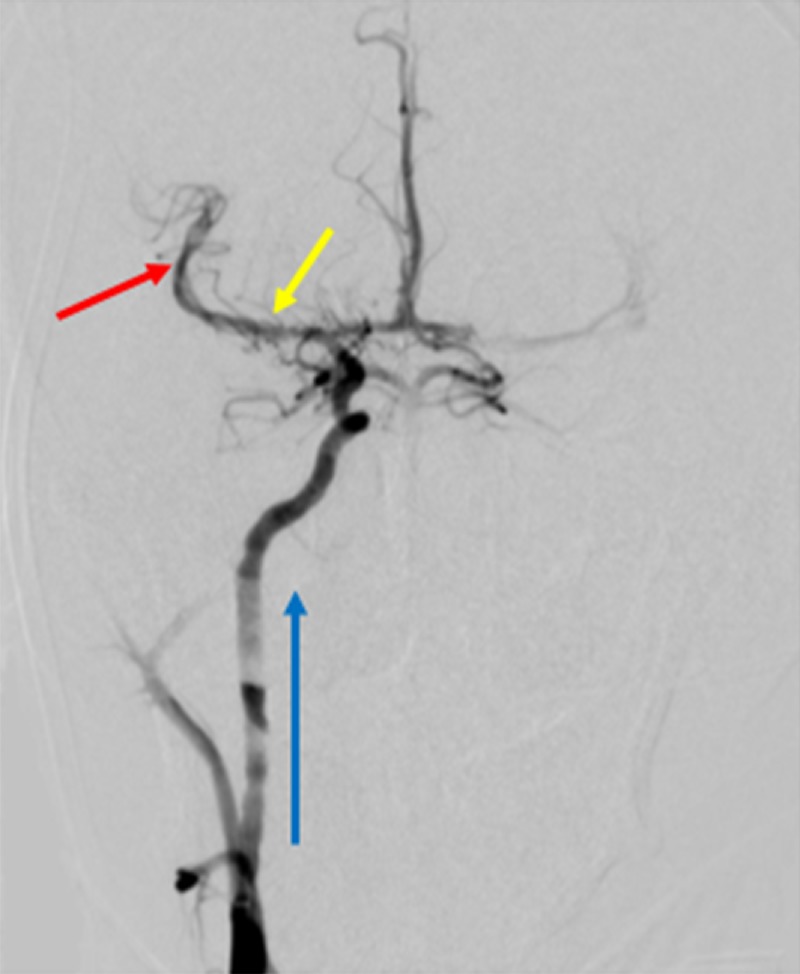
Post-procedural Cerebral Angiogram A post-procedural cerebral angiogram shows complete reperfusion (thrombolysis in cerebral infarction score of three) of the right middle cerebral artery, after a Solitaire® stent-retriever and Penumbra® pump were used to evacuate the thrombus. The blue arrow represents the direction of blood flow from the right internal carotid artery, into the intracranial vasculature. The yellow arrow shows where the thrombus was previously located before reperfusion in the M1 segment. The red arrow shows the reperfused M2 segment after the interventional procedure was completed.

A Solitaire® stent-retriever (Medtronic, Minneapolis, Minnesota) was deployed across the thrombus with an ACE 68® catheter (Penumbra Incorporated, Alameda, California) and advanced to the level of the occlusion. This combination of stent retriever and neurocatheter was left in place for five minutes to allow for engagement of the clot. During this time, the ACE 68® catheter was placed to suction. The stent retriever was then withdrawn with vacuum suction provided by a Penumbra® pump. A follow-up angiogram showed complete reperfusion of the right middle cerebral artery (thrombolysis in cerebral infarction score of three) (Figure 7).

A right femoral arteriogram was performed through the sheath indicating the patient was an adequate candidate for percutaneous access closure. All wires, catheters, and sheaths were removed, and the right groin site was closed with an Angio-Seal® closure device (Terumo Medical Corporation, Somerset, New Jersey). A sterile dressing was placed after hemostasis was ensured, and the patient was transported to the neurocritical care unit.

## Discussion

Steerable microcatheters were developed to allow for added maneuverability and ease of vascular access. Additionally, because of the efficiency of these devices, time to procedure completion is reduced. The article *Welcome to the New Era: A Completely Wireless Interventional Procedure* described the first vascular interventional application of the steerable microcatheter in a patient receiving a uterine artery embolization (UAE) [15]. The time of the procedure was completed in less than half the time allotted for the average radial access UAE, due to the added finesse of a steerable microcatheter and the lack of the need for a guidewire [15]. As seen in the preceding case, a steerable microcatheter was used to assist in rapid reperfusion of a patient presenting with AIS, as was used in previously documented literature to rapidly perform an embolization [15]. The use of a steerable microcatheter in the setting of acuity has been proven to be advantageous, especially in the preceding case of AIS.

There have been other reported uses of the steerable microcatheter in the extravascular setting, though not originally developed for this purpose. Eadie et al. described the use of the first establishment of an otherwise complicated percutaneous nephrostomy, using a steerable microcatheter [16]. Padilla et al. described the first use of a steerable microcatheter to perform a technically challenging cholangiogram when a traditional guidewire would not traverse a tortuous biliary system [17]. The absence of the need for advancement of a steerable microcatheter over a guidewire alone alters the foundation of intervention and the traditional wire and catheter system. As innovational devices become available to operators, procedures will evolve accordingly, ultimately changing the paradigm of interventional management.

Although the preceding case is the first documented demonstration of the efficiency favoring steerable microcatheters in AIS patients, it is foreseen that the application of the steerable microcatheter may become conventional for stroke management in the future. This can be determined by current evidence-based literature, which suggests that mechanical thrombectomy and neuro-intravascular interventions improve patient outcomes after up to 24 hours post-AIS symptom onset [18]. As concluded in the Stryker® Neurovascular DAWN trial, published in February of 2018, CT perfusion or magnetic resonance diffusion-weighted imaging is most sensitive for detecting AIS and acts as the current guidelines for stroke intervention [18]. The Stryker® Neurovascular DAWN trial ultimately advocates for mechanical intervention when possible, in order to salvage what is left of vital brain tissue. Intuitively, the use of steerable microcatheters could be an interesting research endpoint when measuring outcomes in patients presenting with AIS, especially when these novel devices have been shown to eliminate the need for a guidewire and reduce procedure time. 

Though invented for the ease of vascular intervention, the serendipitous application of the steerable microcatheter, encompassing an array of interventional procedures, is telling of both the advancement and refinement of interventional management. Specifically, as in the preceding case, the interventional management of AIS may have improved patient outcomes when steerable microcatheters are used to reperfuse brain parenchyma. Currently, evidence-based data favor the use of interventional management for patients presenting with AIS. This promotes the convention of the steerable microcatheter as a prospectively invaluable device to utilize, more especially when procedure time dictates the outcome of patient prognosis.

## Conclusions

The management of AIS has evolved over the past few decades from the initial discovery of intravenous systemic rtPA to the current guidelines which widely promote the use of neurointerventional management up to 24 hours post-symptom onset. The patient prognosis of AIS has progressively improved with the advancement of devices available to interventional operators. The abundant evidence promoting the use of modern interventional and mechanical thrombectomy devices makes the use of steerable microcatheters for rapid reperfusion in patients with AIS in future felicitous.

## References

[1] Prabhakaran S, Ruff I, Bernstein RA (2015). Acute stroke intervention: a systematic review. JAMA.

[2] National Institute of Neurological Disorders and Stroke rt-PA Stroke Study Group (1995). Tissue plasminogen activator for acute ischemic stroke. N Engl J Med.

[3] Hacke W, Kaste M, Bluhmki E (2008). Thrombolysis with alteplase 3 to 4.5 hours after acute ischemic stroke. N Engl J Med.

[4] Saver JL (2004). Number needed to treat estimates incorporating effects over the entire range of clinical outcomes: novel derivation method and application to thrombolytic therapy for acute stroke. Arch Neurol.

[5] Saver JL, Gornbein J, Grotta J (2009). Number needed to treat to benefit and to harm for intravenous tissue plasminogen activator therapy in the 3- to 4.5-hour window: joint outcome table analysis of the ECASS 3 trial. Stroke.

[6] Jauch EC, Saver JL, Adams Jr HP (2013). Guidelines for the early management of patients with acute ischemic stroke: a guideline for healthcare professionals from the american heart association/american stroke association. Stroke.

[7] González RG, Furie KL, Goldmacher GV (2013). Good outcome rate of 35% in IV-tPA-treated patients with computed tomography angiography confirmed severe anterior circulation occlusive stroke. Stroke.

[8] Broderick JP, Palesch YY, Demchuk AM (2013). Endovascular therapy after intravenous t-PA versus t-PA alone for stroke. N Engl J Med.

[9] Penumbra Pivotal Stroke Trial Investigators (2009). The penumbra pivotal stroke trial: safety and effectiveness of a new generation of mechanical devices for clot removal in intracranial large vessel occlusive disease. Stroke.

[10] Smith WS, Sung G, Saver J (2008). Mechanical thrombectomy for acute ischemic stroke: final results of the multi MERCI trial. Stroke.

[11] Smith WS, Sung G, Starkman S (2005). Safety and efficacy of mechanical embolectomy in acute ischemic stroke: results of the merci trial. Stroke.

[12] Goyal M, Demchuk AM, Menon BK (2015). Randomized assessment of rapid endovascular treatment of ischemic stroke. N Engl J Med.

[13] Berkhemer OA, Fransen PS, Beumer D (2015). A randomized trial of intraarterial treatment for acute ischemic stroke. N Engl J Med.

[14] Goyal M, Demchuk AM, Menon BK (2015). Randomized assessment of rapid endovascular treatment of ischemic stroke. N Engl J Med.

[15] Martin JT, Hulsberg PC, Soule E, Shabandi M, Matteo J (2018). Welcome to the new era: a completely wireless interventional procedure. Cureus.

[16] Eadie E, Harmon TS, Soule E, Hulsberg PC, Shabandi M, Matteo J (2018). A novel nonvascular application of the steerable microcatheter. Cureus.

[17] Padilla RM, Hulsberg PC, Soule E (2018). Against the odds: a novel technique to perform cholangiography from a percutaneous approach through the cystic duct. Cureus.

[18] Nogueira RG, Jadhav AP, Haussen DC (2018). Thrombectomy 6 to 24 hours after stroke with a mismatch between deficit and infarct. N Engl J Med.

